# C-terminus of HSC70-Interacting Protein (CHIP) Inhibits Adipocyte Differentiation via Ubiquitin- and Proteasome-Mediated Degradation of PPARγ

**DOI:** 10.1038/srep40023

**Published:** 2017-01-06

**Authors:** Jung-Hoon Kim, Soyeon Shin, Jinho Seo, Eun-Woo Lee, Manhyung Jeong, Min-sik Lee, Hyun-Ji Han, Jaewhan Song

**Affiliations:** 1 Department of Biochemistry, College of Life Science and Biotechnology, Yonsei University, Seoul, 120-749, Republic of Korea

## Abstract

PPARγ (Peroxisome proliferator-activated receptor γ) is a nuclear receptor involved in lipid homeostasis and related metabolic diseases. Acting as a transcription factor, PPARγ is a master regulator for adipocyte differentiation. Here, we reveal that CHIP (C-terminus of HSC70-interacting protein) suppresses adipocyte differentiation by functioning as an E3 ligase of PPARγ. CHIP directly binds to and induces ubiquitylation of the PPARγ protein, leading to proteasome-dependent degradation. Stable overexpression or knockdown of CHIP inhibited or promoted adipogenesis, respectively, in 3T3-L1 cells. On the other hand, a CHIP mutant defective in E3 ligase could neither regulate PPARγ protein levels nor suppress adipogenesis, indicating the importance of CHIP-mediated ubiquitylation of PPARγ in adipocyte differentiation. Lastly, a CHIP null embryo fibroblast exhibited augmented adipocyte differentiation with increases in PPARγ and its target protein levels. In conclusion, CHIP acts as an E3 ligase of PPARγ, suppressing PPARγ-mediated adipogenesis.

Diabetes mellitus is closely related to other metabolic, cardiac, inflammatory, and osteogenic diseases^1^,^2^,^3^. In particular, glucose intolerance triggered by obesity is one of the major causes of diabetes mellitus and related diseases^4^,^5^. Adipocytes play key roles in maintaining homeostasis of lipids and glucose throughout the body by storing triglycerides and secreting various cytokines and adipokines, including adiponectin and leptin^6^. Thus, understanding the process of adipocyte differentiation is a key step in preventing and overcoming metabolic diseases induced by obesity.

PPARγ is a master transcriptional regulator of adipocyte differentiation, which affects lipid and glucose metabolism^7^,^8^. While its expression is observed in other tissues, PPARγ is predominantly expressed in adipose tissues^9^. PPARγ is both necessary and sufficient for converting pre-adipocytes to adipocytes; this has been demonstrated in PPARγ-knockout mice, which fail to generate adipocytes on a high-fat diet^10^. Because of the clinical and pathological implications in obesity, diabetes, inflammation, and cancer, PPARγ agonists have been developed in an attempt to treat these diseases^11^. The thiozolidindione (TZD) family, including troglitazone, rosiglitazone, and pioglitazone, are the most well-known PPARγ agonists^12^,^13^. However, side effects of these drugs include weight gain and heart and liver failure^14^,^15^. As these side effects prevent the use of TZD-related drugs, non-agonistic compounds capable of regulating PPARγ activation without side effects are currently being sought. Additional research efforts seek to unravel and understand the regulatory mechanisms of PPARγ-related pathways.

C/EBPα (CCAAT/enhancer-binding protein) and PPARγ are co-stimulators, each functioning as a transcription factor to activate the gene expression of the other^16^,^17^,^18^,^19^. Various target genes of PPARγ, such as *aP2* (fatty acid-binding protein), *LPL* (lipoprotein lipase), *CD36*, and *adiponectin* are induced by PPARγ bound to RXR (retinoid X receptor alpha)^9^,^20^. On the other hand, PPARγ function is negatively regulated through direct interaction with its corepressor, NcoR1^21^. Recently, several papers have highlighted the importance of the post-translational modification (PTM) of PPARγ in regulating metabolic disorders^20^,^22^,^23^. For example, MAPK (mitogen-activated protein kinases) and CDK7/9 (Cyclin-dependent kinase7/9) mediate the phosphorylation of PPARγ2 at serine 112, which leads to suppression of adipocyte differentiation^24^. Accordingly, mutation of serine 112 to alanine in a mouse model improves insulin sensitivity in diet-induced obesity^25^. Another well-known phosphorylation site is serine 273, which is activated by CDK5^26^. Mutation of serine 273 in mice led to significant improvements in insulin resistance^26^. Deacetylation and sumoylation of PPARγ by Sirt1 (Sirtuin 1) and Ubc9 (ubiquitin carrier protein 9), respectively, also revealed intricate PTMs that modulate PPARγ regulation^27^,^28^. Furthermore, several E3 ligases, including MRKN1, Siah2, and Nedd4-1, are involved in ubiquitylation of PPARγ^29^,^30^,^31^.

CHIP (C-terminus of HSC70-interacting protein) is an E3 ligase with a variety of target proteins, including p53, PTEN (Phosphatase and tensin homolog), Tau, and RIPK3 (Receptor-interacting serine/threonine-protein kinase 3)^32^,^33^,^34^,^35^. It contains a U-box domain responsible for ubiquitylation activities and a tetratricopeptide repeat (TPR) domain required for protein interactions. In particular, the TPR region is involved in HSP70 (heat shock proteins 70) and Hsp90 (heat shock proteins 90) association^36^. By interacting with molecular chaperones that affect the E3 ligase function in negative or positive ways, CHIP seems to be an essential factor for the maintenance of protein homeostasis. In this paper, we identified a new physiological role of CHIP: regulation of adipocyte differentiation. CHIP induces ubiquitylation and degradation of the PPARγ protein through direct interaction. To confirm this, we show that stable overexpression of CHIP in 3T3-L1 cells suppresses adipocyte differentiation, while CHIP knockdown promotes adipogenesis. In accordance with these observations, CHIP-null mouse embryonic fibroblasts exhibited increased adipocyte differentiation with elevated levels of PPARγ. These results confirm the role of CHIP in suppressing adipogenesis by inducing degradation of the master adipocyte transcription factor PPARγ.

## Results

### CHIP interacts with PPARγ

**Figure 1:**
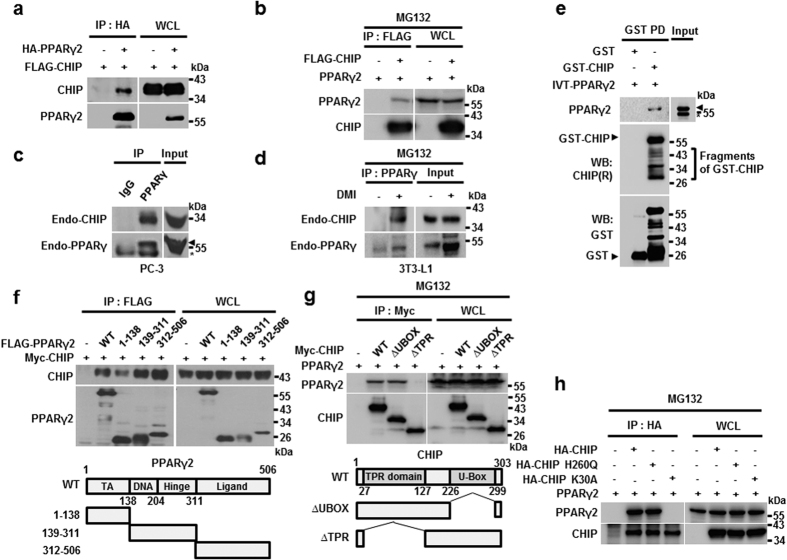
CHIP interacts with PPARγ. (**a**) CHIP binds PPARγ proteins to each other. Lysates of HEK293T cells transfected with pcDNA3-FLAG-CHIP and pcDNA3-HA-PPARγ were immunoprecipitated with α-HA and FLAG antibodies. (**b**) PPARγ binds to CHIP protein reciprocally. HEK293T cells transfected with expressing vector of CHIP and PPARγ were treated with 10 μM MG132 before harvest for 6 hour. (**c–e**) CHIP directly interacts with PPARγ according to endogenous and *in vitro* binding assays. (**c**) PC-3 cells were immunoprecipitated with α-IgG and α-PPARγ, followed by a western blot with polyclonal CHIP and monoclonal PPARγ antibodies. Asterisk indicates a non-specific band. (**d**) Preadipocyte, 3T3-L1 cells were treated with DMI cocktail (Dexamethasone, IBMX and Insulin) during 6 days. Differentiated cells were treated with MG132 for 6 h before harvest. Harvested cells were immunoprecipitated with polyclonal PPARγ antibody. Lysates were analyzed by western blotting indicated antibodies. (**e**) GST- and recombinant GST-tagged CHIP proteins were assessed with GST pull-down assay. Western blot was performed on lysates using monoclonal GST and PPARγ antibodies. Asterisk indicates a non-specific band. (**f,g**) PPARγ binds to the C-terminal region of the CHIP protein, and CHIP binds to the C-terminal region of the PPARγ protein. Lysates of HEK 293 T cells transfected with the indicated plasmids were immunoprecipitated with α-Myc and α-FLAG antibodies, and western blots were performed using the indicated antibodies. (**h**) The CHIP H260Q mutant also binds to the PPARγ protein. Lysates of HEK 293 T cells transfected with HA-CHIP, HA-CHIP H260Q, and FLAG-PPARγ expression vectors were immunoprecipitated with α-HA antibodies, and western blots were performed using the indicated antibodies.

In previous reports, CHIP was found to be capable of modulating the activities of several nuclear receptors, including ARs (Androgen Receptors), ERs (Estrogen Receptors), and GRs (glucocorticoid receptors)^37^,^38^,^39^. This led us to investigate the impact of CHIP on the nuclear receptor PPARγ. We first found that CHIP was able to interact with PPARγ2, a PPARγ isoform, under both exogenous conditions (Fig. 1a,b–d). Endogenous CHIP and PPARγ were also able to bind to each other in both PC-3 and 3T3-L1 cell lines (Fig. 1c,d). Supporting these observations, recombinant GST (glutathione s-transferase)-CHIP was able to bind PPARγ2 that had been transcribed and translated in reticulocyte lysates, indicating a possible direct interaction between the two proteins (Fig. 1e). PPARγ1, another isoform of PPARγ, exhibited similar CHIP-binding capabilities, implying that CHIP may not distinguish between the two isoforms of PPARγ (Supplemental Fig. 1a). To identify the domain responsible for these interactions, we generated several deletion mutants of either PPARγ2 or CHIP and conducted immunoprecipitation analyses. Results showed that CHIP binds to multiple regions of PPARγ2, including DNA- and ligand-binding domains (Fig. 1f). The CHIP TPR region is required for the interaction with PPARγ2 (Fig. 1g). Of note, an E3 ligase-defective mutant, H260Q, was able to bind to PPARγ2, while K30A, a mutant defective in the TPR domain, could not, corroborating the finding that the TPR region is required for the interaction between CHIP and PPARγ2 (Fig. 1h)^40^.

### CHIP is an E3 ligase of PPARγ

**Figure 2:**
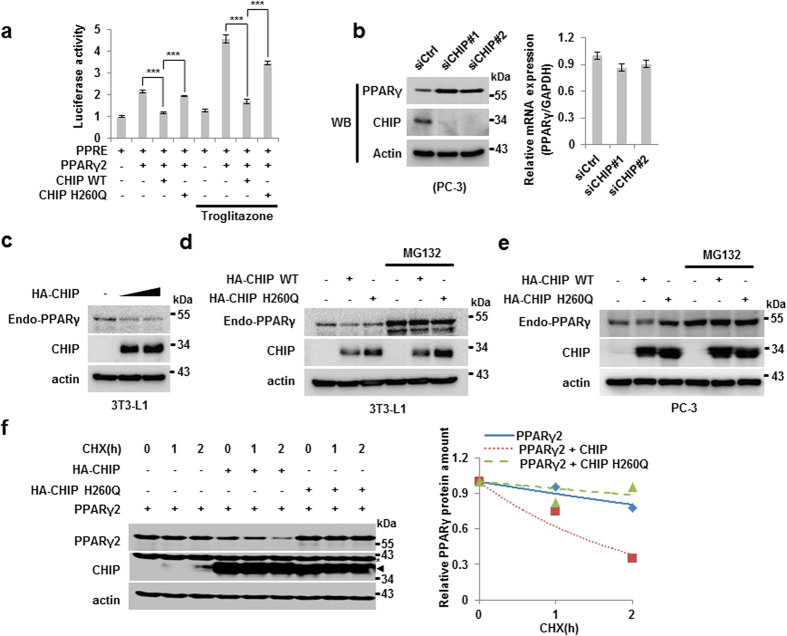
CHIP degrades PPARγ protein through the proteasomal pathway via an E3-ligase function. (**a**) CHIP represses the transcriptional activity of PPARγ. H1299 cells were transfected with PPRE, pcDNA3.1-PPARγ2, pcDNA3-FLAG-CHIP, and pcDNA3-FLAG-CHIP H260Q plasmids with or without troglitazone. Cells were measured by luminometer. Data are presented means ± SD; n = 3; **P < 0.01, and ***P < 0.001 compared to each lane. (**b**) The CHIP protein level was increased by CHIP knockdown using siRNAs. Western blots of lysates of PC-3 cells transfected with the indicated plasmids using siRNA IMAX were performed using the indicated antibodies. (**c**) Endogenous PPARγ protein was degraded by CHIP. Preadipocyte, 3T3-L1 cells were treated with DMI cocktail for 2 days, were transfected with pcDNA3-HA-CHIP plasmid dose dependent manner. Indicated protein was measured by western blotting. (**d,e**) CHIP degrades PPARγ protein through the E3-ligase function and proteasomal manner. (**d**) DMI treated 3T3-L1 cells were transfected with HA-CHIP and HA-CHIP H260Q mutant expressing vectors with or without MG132. Each protein was detected by western blotting indicated antibodies. (**e**) PC-3 cells were transfected with pcDNA3-HA-CHIP and pcDNA3-HA-CHIP H260Q mutant with or without MG132. Lyzed cells were analyzed by western blotting indicated antibodies. (**f**) The PPARγ protein was destabilized by wild-type CHIP but not by the H260Q mutant. Western blots of H1299 cells transfected with plasmids expressing PPARγ, FLAG-CHIP, and FLAG-CHIP H260Q in the presence or absence of 50 μg/mL CHX (cycloheximide) treatment were performed using the indicated antibodies and were measured with the Image J program. Asterisk indicates an actin band.

To observe the effect of CHIP on PPARγ activity, we assessed the activity of a peroxisome proliferator response element (PPRE) promoter-driven luciferase stimulated by PPARγ2 with or without CHIP expression. When PPARγ2 was overexpressed, luciferase activity was doubled, an effect that was inhibited by simultaneous overexpression of CHIP. Importantly, the E3 ligase-defective mutant version of CHIP, H260Q, was not able to suppress PPARγ2 transcription factor activity, implying that post-translational modification processes may be required for the CHIP and PPARγ interaction. The similar effects were observed under the treatment of troglitazone, a artificial ligand of PPARγ (Fig. 2a). The elimination of CHIP using two different CHIP siRNAs in PC3 cells resulted in increased PPARγ protein without altering mRNA levels, indicating that CHIP may in fact regulate PPARγ post-translationally (Fig. 2b).

As CHIP is an E3 ligase and its elimination led to an increase in PPARγ protein, we next tested whether CHIP induced the degradation of PPARγ. Results demonstrated that overexpression of CHIP did indeed induce endogenous and exogenous PPARγ degradation (Fig. 2c and Supplemental Fig. 2a). CHIP-mediated degradation of endogenous PPARγ appears to be proteasome dependent, as degradation is blocked by MG132, a proteasome inhibitor in both 3T3-L1 and PC3 cell lines (Fig. 2d,e). Furthermore, while H260Q, the E3 ligase-defective mutant of CHIP, was able to bind PPARγ2, it was incapable of inducing degradation of endogenous PPARγ, indicating that ubiquitylation is also required (Figs 1h and 2d,e). We observed the similar effects with overexpressed proteins including PPARγ2 and CHIP or H260Q (Supplementary Figure 2b,c). Supporting this, the half-life of PPARγ2 was reduced only by wild-type (WT) CHIP, not H260Q (Fig. 2f). Results for PPARγ1 were similar to those for PPARγ2 (Supplemental Fig. 1b,c). Overall, our results indicate that CHIP may regulate PPARγ via post-translational modification.

**Figure 3:**
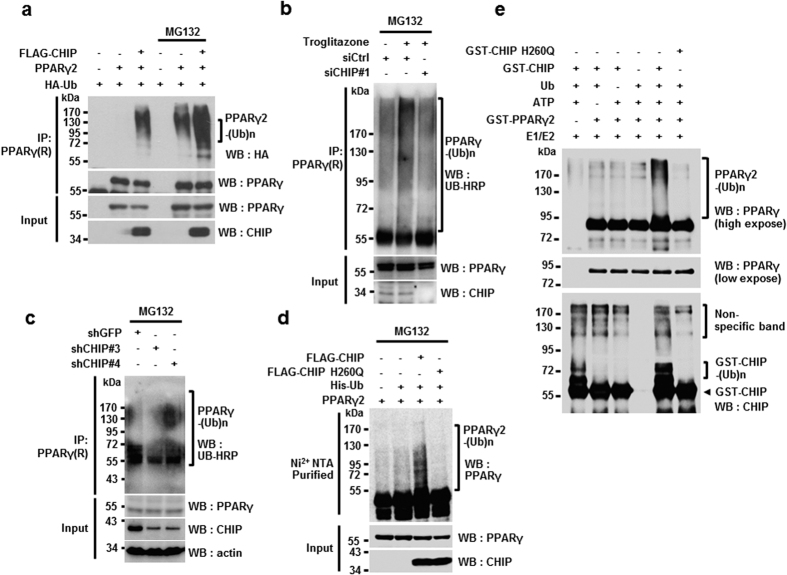
CHIP mediates ubiquitylation of PPARγ2 through its E3 ligase activity. (**a**) CHIP induces ubiquitylation of PPARγ2. Western blots of H1299 cells transfected with pcDNA3.1-PPARγ, pcDNA3.1-FLAG-CHIP, and PMH-HA-Ub with or without MG132 were performed using the indicated antibodies. (**b,c**) Elimination of CHIP did not increase the ubiquitylation of PPARγ. Lysates of PC-3 cells transfected with siRNA control (siCtrl) or siRNA CHIP #1 and treated with MG132 were immunoprecipitated with polyclonal PPARγ antibodies and western blots were performed using the indicated antibodies. (**b**) Lysates of 3T3-L1 cells transfected with CHIP knockdown plasmids expressing shCHIP #3 and shCHIP #4 or control plasmid (shGFP), differentiated by DMI treatment, and treated with MG132 were immunoprecipitated using polyclonal PPARγ. (**c**) Western blots were performed on the above lysates using the indicated antibodies. (**d**) PPARγ2 protein was ubiquitylated by wild-type CHIP but not the H260Q mutant. Western blots of H1299 cells transfected with asdescribe above all, with His-ubiquitin-conjugated proteins pulled down using Ni^2+^-NTA beads. The indicated antibodies were used. (**e**) CHIP directly ubiquitinates PPARγ2 *in vitro*. Western blots of purified GST-PPARγ2 recombinant protein incubated with ATP, E1, E2, and ubiquitin in the presence or absence of wild-type GST-CHIP or GST-CHIP H260Q mutant were performed using the indicated antibodies.

Since CHIP is a well-known E3 ligase, we next examined whether CHIP mediates the ubiquitylation of PPARγ. We found that poly-ubiquitylation of PPARγ2 increased because of CHIP overexpression, with or without MG132 (Fig. 3a). In accordance with these observations, endogenous poly-ubiquitylated forms of PPARγ were reduced by the elimination of CHIP in PC3 cell lines, indicating that CHIP regulates PPARγ endogenously. Here, cells were treated with troglitazone to induce accumulation of PPARγ so that ubiquitylated forms could be detected, as previously reported (Fig. 3b)^29^. As a result, poly-ubiquitylated forms of endogenous PPARγ were also reduced following CHIP knockdown in 3T3-L1 cells that were induced to differentiate into adipocytes by treatment with dexamethasone, IBMX, and insulin (DMI treatment) for two days (Fig. 3c). As with CHIP-mediated degradation of PPARγ2, the E3 ligase activity of CHIP was required for this regulation, as H260Q was not able to induce ubiquitylation of PPARγ2 (Fig. 3d). Finally, using recombinant CHIP, H260Q, and PPARγ2, we confirmed that CHIP directly ubiquitylates PPARγ2 (Fig. 3e). We also observed that CHIP similarly mediates the ubiquitylation of PPARγ1, supporting the conclusion that CHIP induces degradation of PPARγ (Supplemental Fig. 3). In summary, CHIP functions as an E3 ligase by inducing the ubiquitylation and proteasome-dependent degradation of PPARγ.

### HSP70 suppresses CHIP-mediated PPARγ degradation and ubiquitylation

**Figure 4:**
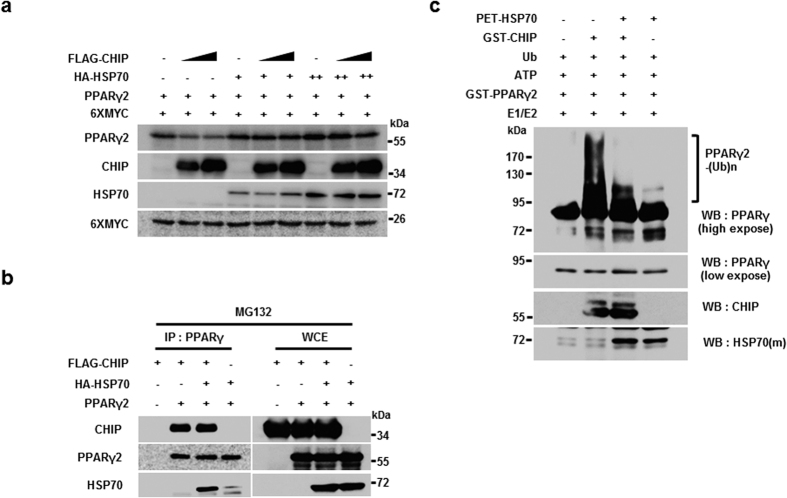
HSP70 suppresses CHIP E3 ligase activity, reducing PPARγ2 degradation. (**a**) CHIP-mediated PPARγ2 degradation was blocked by HSP70. Western blots of H1299 cells transfected with FLAG-CHIP, PPARγ2, 6XMYC, and increasing amounts of HSP70-expressing plasmid were performed using the indicated antibodies. (**b**) Interaction between CHIP and PPARγ2 was not affected by HSP70. HEK 293 T cells were tansfected with pcDNA3-FLAG-CHIP, pcDNA3.1-PPARγ2, and pcDNA3-HA-HSP70 in the presence of MG132 for 6 hour. Lysates of HEK 293 T cells transfected with PPARγ2 and CHIP-expressing plasmids were immunoprecipitated with α-PPARγ antibodies, and western blots were performed using the indicated antibodies. (**c**) HSP70 negatively regulates the E3 ligase function of CHIP, reducing PPARγ2 ubiquitylation. Western blots of purified recombinant proteins (GST-PPARγ2, GST-CHIP, GST-CHIP H260Q, and PET-HSP70) incubated with ATP, E1, E2, and ubiquitin were performed using the indicated antibodies.

Hsp90 and Hsp70 function as major CHIP partners in the regulation of various target proteins^41^,^42^. Ubiquitylation analyses with recombinant proteins in the absence of Hsp70 and Hsp90, however, suggest that these two molecular chaperones may not participate in the CHIP-mediated ubiquitylation of PPARγ (Fig. 3e). To examine the roles of both molecular chaperones in PPARγ degradation processes further, the effect of geldanamycin (GA), an Hsp90 inhibitor, on CHIP-mediated degradation of PPARγ was assessed. Treatment with this inhibitor had little effect on the CHIP-mediated degradation of PPARγ, indicating that HSP90 may not be required for the degradation process (Supplemental Fig. 4). In contrast, transient overexpression of HSP70 inhibited CHIP-mediated degradation of PPARγ (Fig. 4a). While it seems that HSP70 has little effect on the binding affinity between CHIP and PPARγ2, the presence of HSP70 prevents CHIP-mediated PPARγ2 ubiquitylation (Fig. 4b,c). These results indicate that of the two major partners of CHIP, only Hsp70 functions as a negative regulator by suppressing CHIP-mediated ubiquitylation of PPARγ2.

### CHIP suppresses adipocyte differentiation induced by PPARγ activation

**Figure 5:**
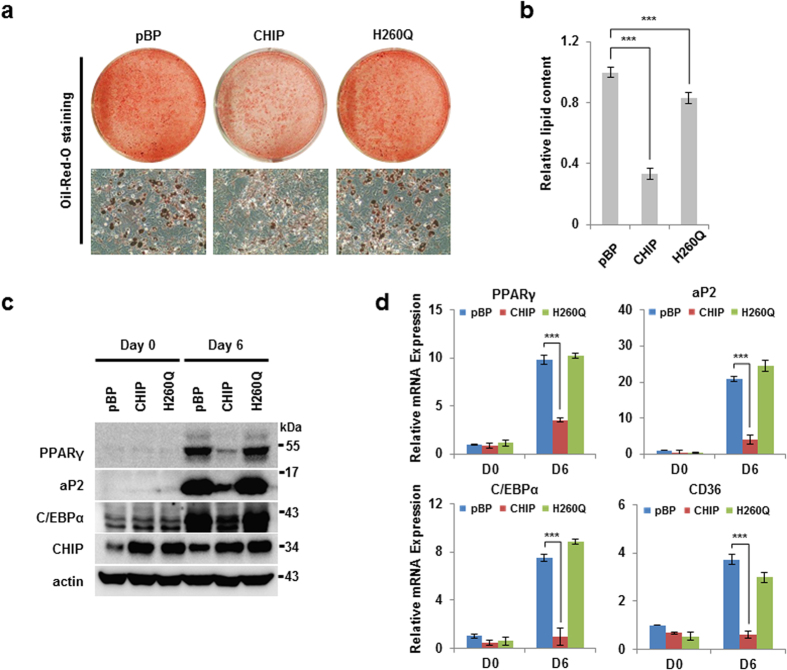
CHIP inhibits adipocyte differentiation in 3T3-L1 cells. (**a,b**) CHIP-overexpressing cells were suppressed during adipogenesis compared to control cells. Preadipocyte 3T3-L1 cells were generated by pBABE puro, pBP-CHIP, and pBP-CHIP H260Q-expressing viral vectors using a virus infection system and were differentiated using a DMI cocktail (dexamethasone, IBMX, insulin). (**a**) Differentiated cells were stained by Oil Red O and were photographed under the microscope. (**b**) Differentiated cells were measured by quantification of lipid content assay using the assay kit. (**c,d**) Protein expression and mRNA of PPARγ included its targets were reduced in CHIP-overexpressing cells. Western blots of lysed differentiated cells were performed using the indicated antibodies and measured by PPARγ or its target mRNA using qRT-PCR. Data are presented means ± SD; n = 3; ***P < 0.001 compared with pBP.

**Figure 6:**
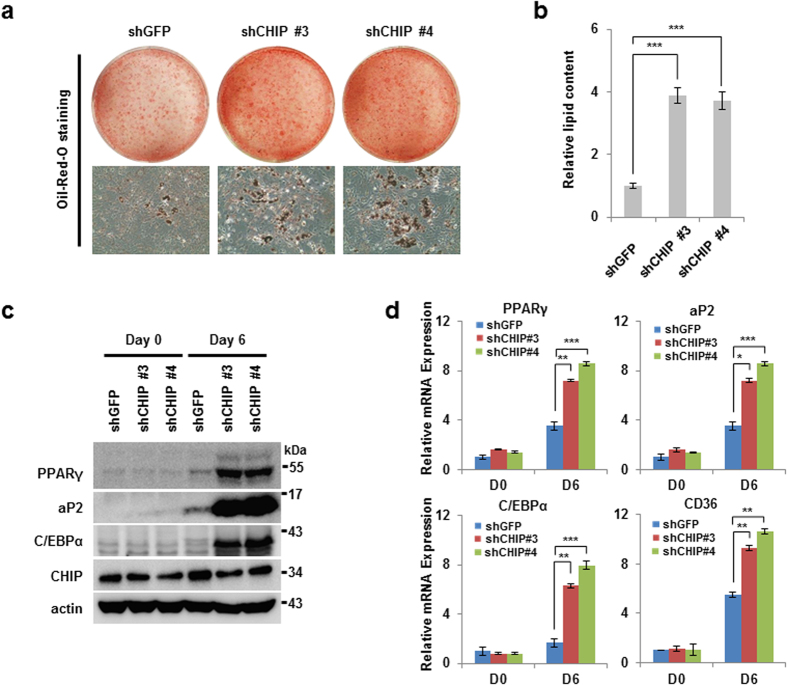
Knockdown of CHIP induces adipocyte differentiation in 3T3-L1 stable cells. (**a**,**b**) Elimination of CHIP accelerates adipocyte differentiation in 3T3-L1 cells. 3T3-L1 cells were infected by CHIP-knockdown lentivirus shRNA (shGFP, shCHIP #3, shCHIP #4) and were differentiated by DI (Dexamethasone and Insulin) cocktail. Differentiated cells were stained and measured as in Fig. 5. (**c**,**d**) PPARγ mRNA and protein levels increased in CHIP-knockdown 3T3-L1 cells. Western blots and qRT-PCR of differentiated cells were performed using the indicated antibodies and primers. Data are presented means ± SD; n = 3; *P < 0.05, **P < 0.01, and ***P < 0.001 compared with shGFP.

**Figure 7:**
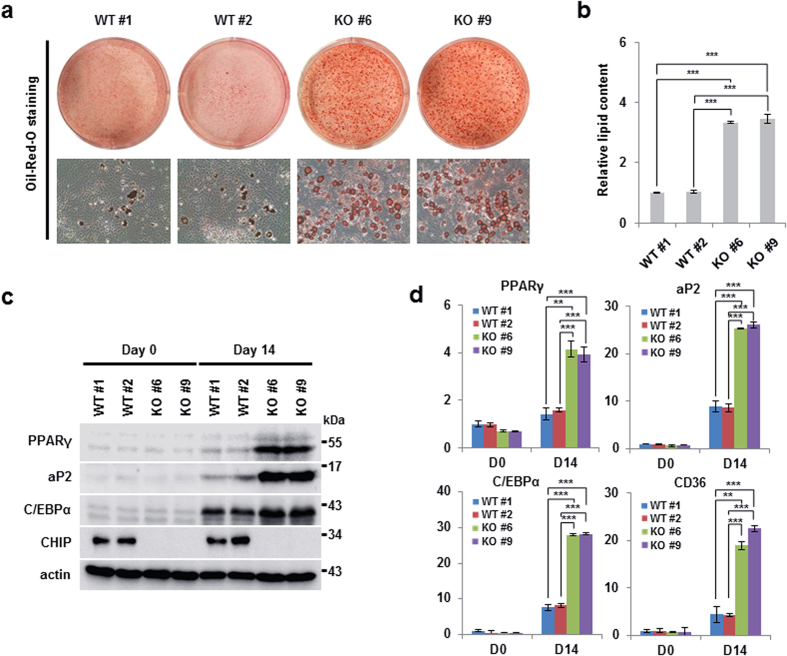
Elimination of CHIP suppresses adipogenesis in mouse embryo fibroblast (MEF) cells. (**a,b**) CHIP-knockout MEFs were induced to undergo accelerated adipocyte differentiation. CHIP-knockout MEFs generated through the instrumental method were induced to undergo differentiation into adipocytes through treatment with DMI and the PPARγ ligand troglitazone. (**a**) Differentiated cells were stained using Oil Red O and were photographed as described in Fig. 5. (**b**) Lipid content was analyzed by quantification kit in differentiated cells. (**c,d**) PPARγ protein and mRNA levels were elevated in CHIP-knockout MEFs. (**c**) Western blots of differentiated MEFs were performed using the indicated antibodies. (**d**) Messenger RNA of PPARγ and its targets was detected by qRT-PCR using the indicated primers. Data are presented means ± SD; n = 3; **P < 0.01, and ***P < 0.001 compared to each lane.

Because CHIP destabilizes the PPARγ protein via ubiquitylation and proteasome-dependent degradation, we next tested whether CHIP is able to regulate adipocyte differentiation in 3T3-L1 cells. First, we generated 3T3-L1 cell lines that stably overexpress CHIP or H260Q using a retroviral system. Along with a control cell line transfected with pBABE, these cell lines were induced to differentiate into adipocytes through DMI treatment. Oil Red O staining data and quantification of lipid content showed that the cell line overexpressing CHIP displayed reduced levels of adipogenesis compared to both the control and H260Q-transfected cells (Fig. 5a,b). We next examined the protein and mRNA levels of PPARγ and its targets in the same cell lysates. As expected, cells overexpressing CHIP exhibited reductions in both PPARγ mRNA and protein levels. We also observed reductions in the PPARγ targets aP2, cofactor C/EBPα and CD36 in cells overexpressing CHIP (Fig. 5c–d). We further generated 3T3-L1 cell lines with stable knockdown of CHIP (shCHIP#3, shCHIP#4) using a lentiviral vector system. In contrast to the overexpression system, stable cell lines with CHIP knockdown displayed more adipocyte differentiation than that of control cells (shGFP) detected by Oil Red O staining (Fig. 6a), and this was confirmed by quantification of lipid content (Fig. 6b). The cell lines expressing shCHIP#3 and shCHIP #4 were further analyzed by western blot and qRT-PCR. Like PPARγ, aP2, C/EBPα and CD36 were elevated following CHIP knockdown when compared to control cells (Fig. 6c,d). Finally, WT or CHIP-null MEFs were employed to examine the effects of CHIP on adipocyte differentiation induced by DMI plus troglitazone, a PPARγ ligand^29^. Two CHIP knockout MEFs, CHIP KO #6 and CHIP KO #9, exhibited greater adipocyte differentiation than the two wild-type MEFs, WT #3 and #8 (Fig. 7a). The data showing quantification of lipid contents confirmed these results (Fig. 7b). Similarly, mRNA or protein levels of PPARγ, aP2, C/EBPα and CD36 were all elevated in CHIP KO #6 and CHIP KO #9 MEFs compared with those of controls (Fig. 7c,d). In summary, these data suggest that CHIP functions as a negative modulator of adipocyte differentiation in 3T3-L1 and primary MEF cells, possibly by suppressing PPARγ.

## Discussion

In this study, we identified a new role of CHIP in adipocyte differentiation. CHIP interacts with and mediates the ubiquitylation of PPARγ, which results in negative effects on adipogenesis. CHIP is commonly known to exert its E3 ligase effects in association with the molecular chaperones Hsp90 and Hsp70^43^. For example, Hsp70 facilitates the CHIP-mediated degradation of p53, HIF-1α, and AR^32^,^37^,^41^, while Hsp90 is known to suppress CHIP-mediated degradation by promoting the stability of CHIP target proteins^44^,^45^. Notably, PPARγ degradation induced by CHIP does not seem to require the presence of either Hsp90 or Hsp70. The poly-ubiquitylation of PPARγ occurs independently of the molecular chaperones, as demonstrated by ubiquitylation analyses with recombinant proteins. The inhibition of Hsp90 by geldanamycin does not have a strong effect on CHIP-mediated PPARγ degradation. Interestingly, it has been reported that Hsp90 inhibition leads to suppression of adipogenesis^46^. Based on our results, it appears that Hsp90 may regulate adipocyte differentiation via a CHIP-independent regulatory pathway. In contrast, Hsp70 appears to function as a negative regulator of CHIP-dependent PPARγ degradation. Upon expression of Hsp70, both the ubiquitylation and degradation of PPARγ were suppressed. A similar pathway, in which Hsp70 functions as a negative regulator of CHIP, can be found in the CHIP-dependent degradation of Tau^34^. In this case, CHIP-mediated ubiquitylation of Tau is suppressed by Hsp70. Since Hsp70 suppresses the function of CHIP, it may function as a positive regulator in adipogenesis by stabilizing PPARγ. Further physiological studies on the role of Hsp70 in adipocyte differentiation and its systemic effects should be conducted in the future.

Several other E3 ligases have been previously reported to ubiquitylate PPARγ. MKRN1 was the first E3 ligase known to mediate PPARγ ubiquitylation and degradation^29^. SIAH2 and NEDD4-1 were subsequently identified as E3 ligases of PPARγ^30^,^31^. With the addition of CHIP based on our results, it appears that PPARγ may be regulated by several E3 ligases. However, most of these observations occurred while investigating cellular differentiation. To determine the role of CHIP in other physiological settings, we generated CHIP-null MEFs and compared differentiation in WT and CHIP-null MEFs. The results demonstrated that CHIP-null MEFs differentiated into adipocytes more effectively than WT MEFs, supporting our *in vitro* data (Figs 1, 2, 3). Based on these observations, the systemic effects of CHIP should be further assessed in the future. Since the whole-body CHIP knockout mouse of the C57BL/6 strain does not survive for more than one month, a conditional knockout system must be established to investigate the physiological roles of CHIP in association with PPARγ^35^,^47^. Additional studies should examine whether the E3 ligases identified so far are expressed in adipocyte tissues and whether their expression levels are regulated by a high-fat diet. Any E3 ligase whose expression is negatively correlated with that of PPARγ under high-fat diet conditions may be a good candidate for drug targeting^48^. Further *in vivo* studies utilizing high-fat-diet induced mice may indicate the main E3 ligase of PPARγ.

TZD and its analogs have been clinically tested for treating obesity and diabetes mellitus through the agonistic activation of PPARγ^49^,^50^. Unfortunately, these drugs exhibited various side effects, including heart and liver failure, weight gain, fluid retention, and bone fractures^51^,^52^. As these drugs have been withdrawn from the market or removed from general usage due to their severe side effects, the concept of developing novel, non-agonistic drugs has been pursued^53^. As various post-translational modifications could regulate the function of PPARγ in possibly non-agonistic ways, targeting post-translational regulators may be a good strategy for discovering non-agonistic drugs with reduced side effects^54^. Since the suppression of CHIP may stabilize and activate PPARγ, CHIP suppression may represent a good target for clinical trials for the treatment of diabetes and obesity.

## Methods

### Adipocyte differentiation

Pre-adipocyte 3T3-L1 cells were used to measure adipocyte differentiation. When cells were almost confluent, differentiation was induced with DMEM (dulbecco’s modified eagle’s medium, Corning Cellgro) containing 10% FBS (fetal bovine serum), 1% penicillin/streptomycin, IBMX (3-isobutyl-1-methylxanthine) (520 μM), insulin (1 μg/mL), and dexamethasone (1 μM) (DMI treatment) for two days. The medium was changed to DMEM containing insulin for an additional two days. On day 4, further differentiation was induced by changing the medium to FBS only. MEF cells were generated as previously described^29^. In the case of adipocyte differentiation of MEF cells, troglitazone was added as a PPARγ ligand during each step. Fully differentiated adipocyte cells were detected under a microscope by Oil Red O staining. Stained cells were extracted with isopropanol. Extracted stains were measured by microplate reader for quantification of differentiation density.

### Cell culture

H1299, 293FT, and MEF cells were maintained in DMEM containing 10% FBS (Corning Cellgro) and 1% penicillin/streptomycin (Gibco, NY, USA). PC-3 cells were cultured in RPMI (Hyclone) supplemented with 10% FBS (Corning Cellgro). NIH 3T3-L1 cells were maintained in DMEM (Gibco) with 10% calf bovine serum (Gibco) and 1% penicillin/streptomycin (Gibco). We purchased the 293FT cell line from Invitrogen, and the 3T3-L1 and H1299 cell lines were purchased from ATCC (American Type Culture Collection). PC-3 cells were provided by Kyung-sup Kim (Yonsei University College of Medicine, Seoul).

### Generation of mouse fibroblast cells

All of mouse experiments were approved by The Institutional Animal Care and Use Committees of Yonsei University (IACUC-A-201409-294-01). The mice were maintained in accordance with the approved guidelines and regulations for experimental animals provided by Yonsei Laboratory Animal Research Center. Using C57BL/6 J mice, heterogeneous CHIP male and female mice were mated to produce wild type and knockout CHIP mice, as previously reported^35^. Mouse embryos were obtained 13.5 days after verifying plug formation. Embryos were minced with a blade in 1 ml trypsin-EDTA. Cells were transferred to 150-mm plates containing DMEM medium with 10% FBS. After 16 hours, cells were maintained in DMEM medium.

### Plasmids

The following constructs were used: pcDNA3.1-PPARγ1, pcDNA3.1-PPARγ2, pCS4-3xHA-PPARγ2, pCS4-3xFLAG-PPARγ2, pGEX-4T1-PPARγ2, pTK-PPREx3-luc, pBabe-puro, pcDNA3-His-Ub, and pHM6-HA-Ub^29^. In addition, we used the following CHIP plasmids, which have been previously described^35^: pcDNA3-FLAG-CHIP, pcDNA3-FLAG-CHIP H260Q, pcDNA3-MYC-CHIP, pcDNA3-MYC-CHIP ∆UBOX, pcDNA3-MYC-CHIP ∆TPR, pGEX-4T1-CHIP, and pGEX-4T1-CHIP H260Q. Other CHIP constructs (pcDNA3-HA-CHIP, pcDNA3-HA-CHIP H260Q, and pcDNA3-HA-CHIP K30A) were sub-cloned from pcDNA3-FLAG-CHIP. An HA-HSP70 expressing plasmid (pcDNA3-HA-HSP70) was generated by polymerase chain reaction followed by insertion into pcDNA3-HA. The PET-HSP70 plasmid has been described previously^55^.

### Antibodies and chemicals

The following antibodies were used in this study for western blots and immunoprecipitation assays: anti-CHIP (rabbit C3B6; Cell Signaling Technology, Danvers, Massachusetts, USA), MYC (sc-40; Santa Cruz Biotechnology, CA, USA), FLAG (mouse F3165; Sigma-Aldrich, St. Louis, MO, USA), FLAG (rabbit; Sigma-Aldrich), HA (mouse sc-7392; Santa Cruz Biotechnology, CA, USA), PPARγ (mouse sc-7273X, Santa Cruz Biotechnology), PPARγ (rabbit sc-7196X; Santa Cruz Biotechnology), aP2 (goat sc-18661; Santa Cruz Biotechnology), C/EBPα(rabbit sc-61X; Santa Cruz Biotechnology), β-actin (A5316; Sigma-Aldrich), MG132 (M-1157, A.G.Scientific, San Diego, CA, USA), and geldanamycin (9843 S, Cell Signaling Technology). Pepstatin A (P5318), aprotinin (A1153), phenylmethanesulfonylfluoride (PMSF; P7626), leupeptin (L2884), dimethyl sulfoxide (DMSO; D8418), N-ethylmaleimide (NEM; E3876), dexamethasone (D1756), 3-isobutyl-1-methylxanthine (IBMX; I5879), and Oil Red O (O0625) were purchased from Sigma-Aldrich (St. Louis, MO, USA), while insulin (11 376 497 001) was purchased from Roche (Mannheim, Germany).

### Transfection

Lipofectamine 2000 (Invitrogen, Carlsbad, CA, USA) was used to transfect 293FT and H1299 cells according to the manufacturer’s instructions. PC-3 cells were transfected with the siRNAs CHIP #1 (5′-CCA GCT GGA GAT GGA GAG TTA-3′) and CHIP #2 (5′-CTG CGC GGG CTG CGC GCT CTA-3′) using Lipofectamine RNAi MAX (Invitrogen). Preadipocyte, 3T3-L1 cells were transfected with the plasmids using Viafect (Promega, Woods Hollow Road Madison, WI, USA, E4981).

### Quantitative RT-PCR analysis

Extraction of RNA was performed using Trizol (Invitrogen) according to the manufacturer’s instructions. Total cDNA was synthesized from isolated RNA using M-MLV reverse transcriptase (Takara Bio Company, Otsu, Japan). Samples were examined by quantitative PCR using a QuantiTect SYBR Green PCR Kit (Qiagen, CA, USA) and the following primers: glyceraldehyde 3-phosphate dehydrogenase (GAPDH; 5′-GGC TGC TTT TAA CTC TGG TA-3′ and 5′-ACT TGA TTT TGG AGG GAT CT-3′), mouse PPARγ (5′-CCA TTC TGG CCC ACC AAC-3′ and 5′-AAT GCG AGT GGT CTT CCA TCA-3′), human PPARγ (5′-TTC AGA AAT GCC TTG CAG TG-3′ and 5′-CCA ACA GCT TCT CCT TCT CG-3′), aP2 (5′-CAC CGC AGA CGA CAG GAA G-3′ and 5′-GCA CCT GCA CCA GGG C-3′), 36B4 (5′-AGA TGC AGC AGA TCC GCA T-3′ and 5′-GTT CTT GCC CAT CAG CAC C-3′), and C/EBP-α (5′-GCG GGC AAA GCC AAG AA-3′ and 5′-GCG TTC CCG CCG TAC C-3′). The expression level of each gene was determined from 3T3-L1 and MEF cell samples and was normalized to the expression of the 36B4 gene in the same sample. The expression levels of genes in PC-3 cells were normalized to those of GAPDH.

### Ubiquitylation assays

H1299 cells were transfected with HA-tagged ubiquitin (PRK5-HA-ub), pcDNA3.1, and pcDNA3.1-PPARγ plasmids with or without pcDNA3-FLAG-CHIP and pcDNA3-FLAG-CHIP H260Q in the presence of MG132. Using PBS containing NEM (10 nM), harvested cells were lysed by boiling in 1% SDS for 10 min. Lysates were produced by adding protease inhibitors (2 μM leupeptin, 1 μM pepstatin A, 2 μM aprotinin, 200 μM PMSF) and lysis buffer with NEM to approach 0.1% SDS concentration. Samples were immunoprecipitated with monoclonal α-PPARγ antibody and were analyzed by western blot using the indicated antibodies. To assess ubiquitylation using Ni^2+^-NTA beads, H1299 cells were transfected with His-tagged ubiquitin (pcDNA3-His-Ub), pcDNA3.1-PPARγ, pcDNA3-FLAG-CHIP, and pcDNA3-FLAG-CHIP H260Q in the presence of MG132. Cells were harvested with NEM containing PBS and were lysed by 6 M guanidine-sodium phosphate buffer (pH 7). Samples were combined with Ni^2+^-NTA beads for 4 hours and were eluted by 200 mM imidazole. Western blotting was performed as described above. Endogenous ubiquitylation was analyzed in PC-3 and 3T3-L1 cells. PC-3 cells were transfected with siRNA CHIP #1 for 24 hours and were then treated with troglitazone for an additional 12 hours to induce endogenous PPARγ ubiquitylation. In 3T3-L1 cells, CHIP was stably knocked down with shCHIP#3 and shCHIP#4, and differentiation was induced with DMI for two days before cells were treated with MG132 for six hours. Harvested and lysed cells were immunoprecipitated with polyclonal α-PPARγ antibody and were measured by western blot using the indicated antibodies. For *in vitro* ubiquitylation, recombinant proteins (GST-PPARγ2, GST-CHIP, and GST-CHIP H260Q) were incubated with 10 μg ubiquitin (Sigma, U6235), 2 mM ATP (Fermentas, R0441), 200 ng E1 (UBE1, Boston Biochem, E-305), and 500 ng E2 (UbcH5c, Boston Biochem, E2-627) in reaction buffer (40 mM Tris-HCL pH 7.6, 50 mM NaCl, and 1 mM DTT) for four hours at 37 °C. Samples were boiled and diluted with buffer to 0.1% SDS and then were immunoprecipitated using monoclonal α-PPARγ antibody. Ubiquitylation of PPARγ was detected by western blot, as described above.

### Protein purification, *in vitro* translation, and *in vitro* binding assays

pGEX-4T-1-CHIP, pGEX-4T-1-CHIP H260Q, and pGEX-4T-1-PPARγ2 were expressed in *Escherichia coli* and purified using glutathione sepharose 4B (GE Healthcare, UK). Bacteria expressing GST-tagged proteins (GST- PPARγ2, GST-CHIP, and GST-CHIP H260Q) were lysed using lysis buffer (300 mM NaCl, 25 mM sodium phosphate buffer at pH 7, 20 mM beta-mercaptoethanol, protease inhibitors, and 5% glycerol). GST-PPARγ2, GST-CHIP, and GST-CHIP H260Q were purified using GST beads (GE Healthcare, UK) following the manufacturer’s protocol. A His-HSP70-expressing vector (PET-HSP70) was examined via transformation into BL21 cells. Cells were lysed using Tris-HCl buffer (50 mM NaCl, 40 mM Tris-HCl at pH 7, 20 mM beta-mercaptoethanol, protease inhibitors, and 5% glycerol). Lysed protein was purified using Ni^2+^-NTA beads and was eluted using 250 mM imidazole. A TNT T7-coupled reticulocyte lysate system (L4610, Promega, USA) was used to produce PPARγ2 protein. Translated protein was incubated with GST or GST-PPARγ2 for two hours and then with sepharose beads for one hour. Complexes were then washed and eluted with reduced glutathione (10 mM), followed by western blot analysis.

### Generation of CHIP stable cell lines

CHIP overexpression cell lines were generated using pBABE-puro-CHIP and pBABE-puro-CHIP H260Q plasmids and the packaging cell line HEK-293FT, as previously described^35^. All shRNA constructs were purchased from Sigma-Aldrich (St. Louis, MO, USA) with the following sequences: mouse CHIP#3 (5′-CACGATAAATACATGGCAGAT-3′) and CHIP#4 (5′-GAGAGTGAGCTGCATTCATAT-3′). NIH-3T3-L1 cells stably expressing shGFP or mouse CHIP shRNA (shCHIP#3 and shCHIP#4) were established for generation of CHIP knockdown cell lines using the manufacturer’s instructions. We transfected 293FT cells with lentiviral packaging vectors and pLKO.1-shGFP or shCHIP#3/#4. At 48 h post-transfection, viral supernatants were harvested, filtered, and added to 3T3-L1 cells. Infected 3T3-L1 cells were selected by puromycin treatment for seven days.

### Quantification of lipid content

The lipid contents were measured with triglyceride quantification colorimetric/fluorometric kits (Biovision, Milpitas, CA, K622-100). Analysis of lipid contents was performed by Biovision as described. Differentiated cells were collected in PBS. Cells were homogenized with 5% NP-40 diluted with water. Lysates and triglycerides were slowly boiled at 80 to 100 °C in a water bath and then cooled down to room temperature. Triglyceride standards were used to make a standard curve. Lysed samples were incubated with lipase in the assay buffer for 20 min, and a mixture of the triglyceride probe was added to the buffer, followed by incubation for 30 to 60 min. The absorbance of the samples was measured with a microtiter plate reader at 570 nm. The values were entered into the provided formula to obtain the final results.

### Statistical analyses

Statistical analyses were conducted using Prism (GraphPad Software Inc., CA, USA), and results are presented as means ± SD. All statistical results in Prism were performed using unpaired two-tailed t-test to compare two groups (n ≥ 3).

## Additional Information

**How to cite this article:** Kim, J.-H. *et al*. C-terminus of HSC70-Interacting Protein (CHIP) Inhibits Adipocyte Differentiation via Ubiquitin- and Proteasome-Mediated Degradation of PPARγ. *Sci. Rep.* **7**, 40023; doi: 10.1038/srep40023 (2017).

**Publisher's note:** Springer Nature remains neutral with regard to jurisdictional claims in published maps and institutional affiliations.

## Supplementary Material

Supplementary Information
