# Supplementary material for: C-terminus of HSC70-Interacting Protein (CHIP) Inhibits Adipocyte Differentiation via Ubiquitin- and Proteasome-Mediated Degradation of PPARγ
Source: Sci Rep. 2017 Jan 6;7:40023. doi: 10.1038/srep40023 (PMC5216347; doi:10.1038/srep40023)
Supplement: Supplementary Information [file srep40023-s1.pdf]

C-terminus of HSC70-Interacting Protein (CHIP) Inhibits Adipocyte Differentiation via Ubiquitin- and Proteasome-Mediated Degradation of PPARγ

Jung-Hoon Kim, Soyeon Shin, Jinho Seo, Eun-Woo Lee, Manhyung Joeng, Min-sik Lee, Hyun-Ji Han, Jaewhan Song*

Department of Biochemistry, College of Life Science and Biotechnology, Yonsei University, Seoul, 120-749

*Corresponding author: J. Song, Department of Biochemistry, Yonsei University, Yonsei-ro 50, Seodaemun-gu, Seoul 120-749, Republic of Korea; E-mail: jso678@yonsei.ac.kr; Tel.: +82 2 2123 5695; Fax: +82 2 362 9897

Supplementary information

Supplementary Figure Legend

Supplementary Figure 1. PPARγ1 is regulated by CHIP. (**a**) PPARγ1 binds to the CHIP protein. Lysates of HEK 293T cells transfected with pcDNA3.1-PPARγ1 and pcDNA3 FLAG-CHIP plasmids in the presence of MG132 were immunoprecipitated with α-FLAG antibodies, and western blots were performed using α-FLAG and α-PPARγ antibodies. (**b**) PPARγ1 protein is degraded by CHIP protein. Western blots of H1299 cells transfected with FLAG-CHIP, FLAG-CHIP H260Q, PPARγ1, and 6XMYC expressing vectors were performed using the indicated antibodies. (**c**) PPARγ1 is destabilized by CHIP. Western blots of H1299 cells transfected with FLAG-CHIP, FLAG-CHIP H260Q, and PPARγ1-expressing plasmids with CHX for the indicated length of time (0, 1, or 2 hours) were performed using the indicated antibodies.

Supplementary Figure 2. Exogenous PPARγ2 protein was regulated by CHIP through the proteasomal degradation. (**a**) PPARγ2 protein was degraded by CHIP. Western blots of H1299 cells transfected with pcDNA3.1-PPARγ2 (0.3 μg), 6XMYC (0.3 μg), and increasing concentrations of FLAG-CHIP (0.2, 0.3, and 0.4 μg) were performed using α-FLAG, α-PPARγ, and α-MYC antibodies. 6XMYC was used as a transfection control. (**b**) CHIP-mediated degradation of PPARγ was blocked by a proteasome inhibitor. Western blots of lysates from H1299 cells transfected with the indicated plasmids and treated with or without MG132, a proteasome inhibitor were performed using the indicated antibodies. (**c**) The CHIP H260Q mutant does not degrade the PPARγ protein. Western blots of H1299 cells transfected with the indicated plasmids were performed with the indicated antibodies.

Supplementary Figure 3. PPARγ1 is ubiquitylated by CHIP. CHIP ubiquitinates PPARγ1 using the E3 ligase function. Lysates of H1299 cells transfected with pcDNA3-FLAG-CHIP, pcDNA3-FLAG-CHIP H260Q, PRK5-HA-Ub, and pcDNA3.1-PPARγ1 in the presence of MG132 were immunoprecipitated with α-PPARγ antibodies, and western blots were performed using the indicated antibodies.

Supplementary Figure 4. HSP90 does not affect CHIP-mediated PPARγ degradation. (a) Western blots of H1299 cells transfected with pcDNA3.1-PPARγ, pcDNA3-HA-CHIP, pcDNA3-HA-CHIP H260Q, pcDNA3-HA-CHIP K30A, and PBEG-GST plasmids with or without 100 nM GA during 20 hour were performed using the indicated antibodies. GST was used as a transfection control.

Supplementary Figures

Sup. Fig. 1

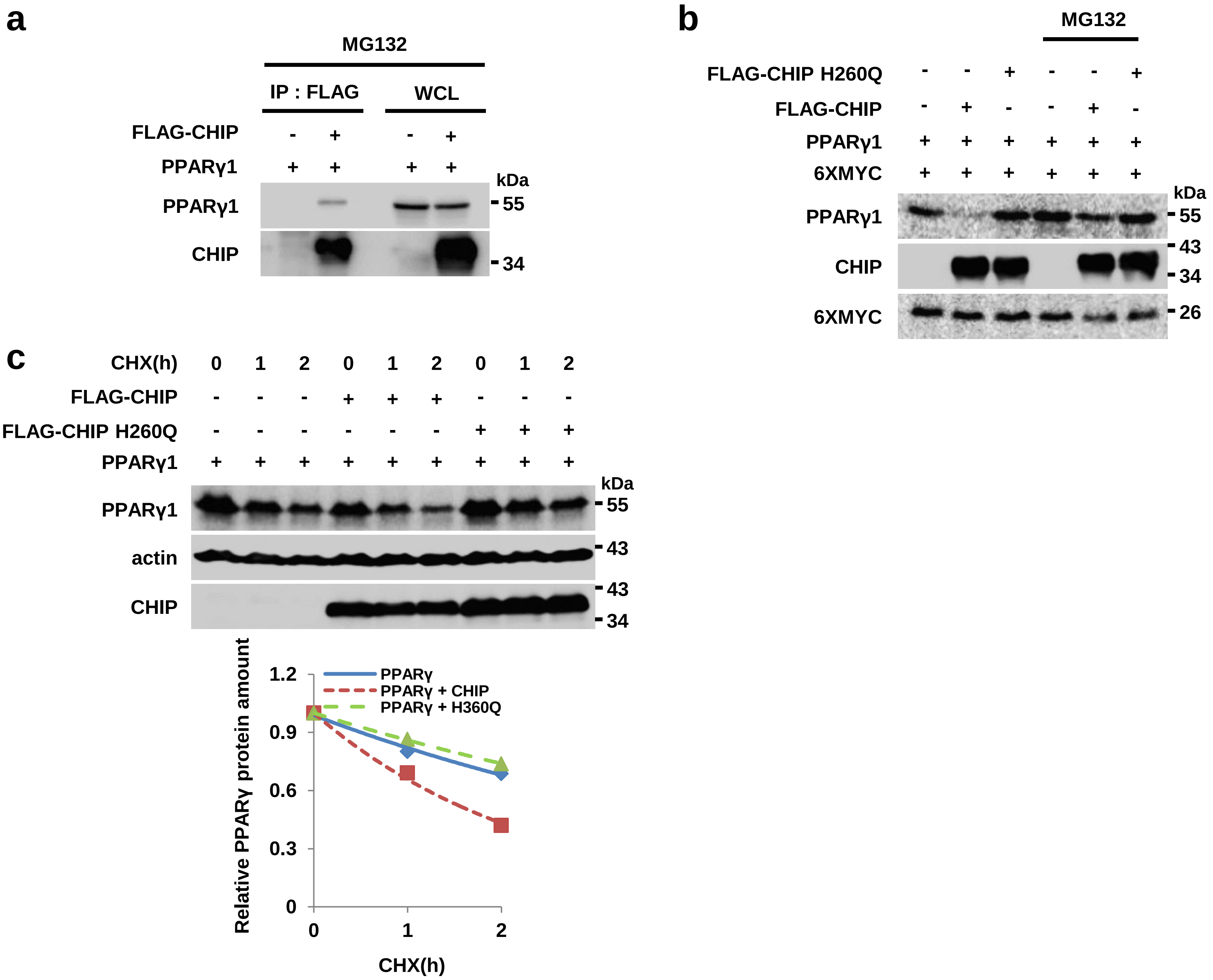

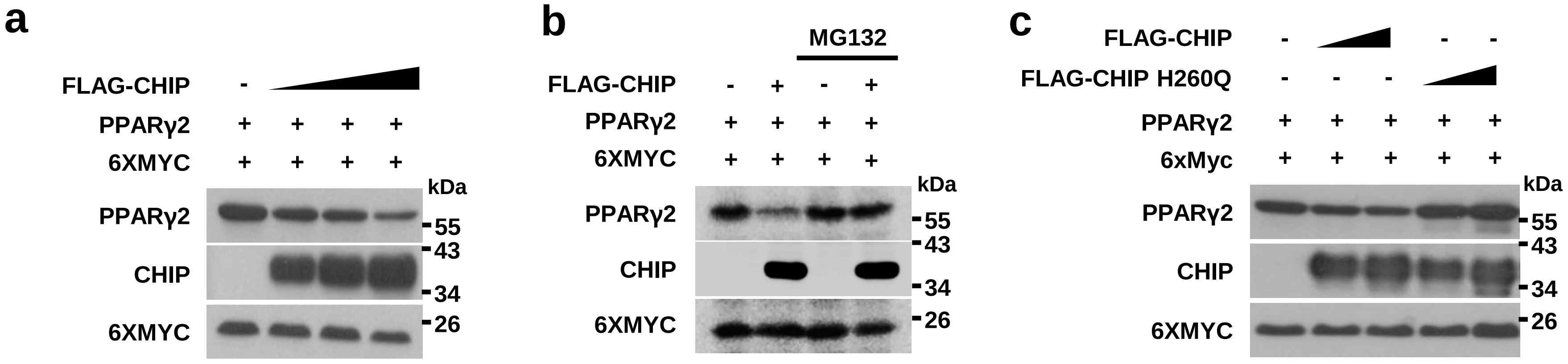
a
FLAG-CHIP
-
PPARγ2
+ + + +
6XMYC
+ + + +
kDa
PPARγ2
55
CHIP
43
34
6XMYC
26
b
MG132
FLAG-CHIP
- + - +
PPARγ2
+ + + +
6XMYC
+ + + +
kDa
PPARγ2
55
CHIP
43
34
6XMYC
26
c
FLAG-CHIP
- - -
FLAG-CHIP H260Q
- - -
PPARγ2
+ + + + +
6xMyc
+ + + + +
kDa
PPARγ2
55
CHIP
43
34
6XMYC
26

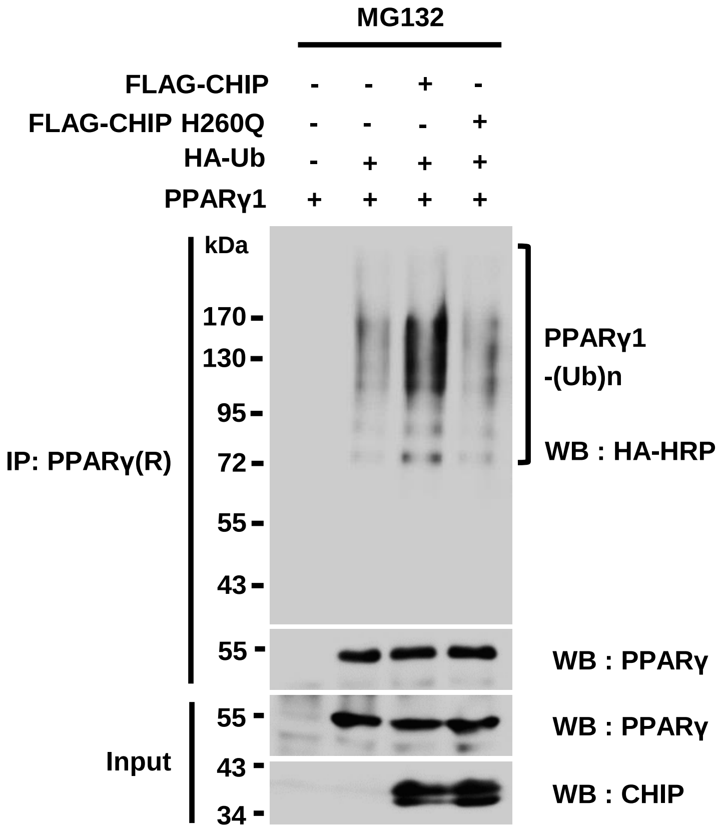
MG132
FLAG-CHIP - - + -
FLAG-CHIP H260Q - - - +
HA-Ub - + + +
PPARγ1 + + + +
kDa
170
130
95
72
55
43
IP: PPARγ(R)
PPARγ1
-(Ub)n
WB : HA-HRP
55
WB : PPARγ
Input
55
WB : PPARγ
43
34
WB : CHIP

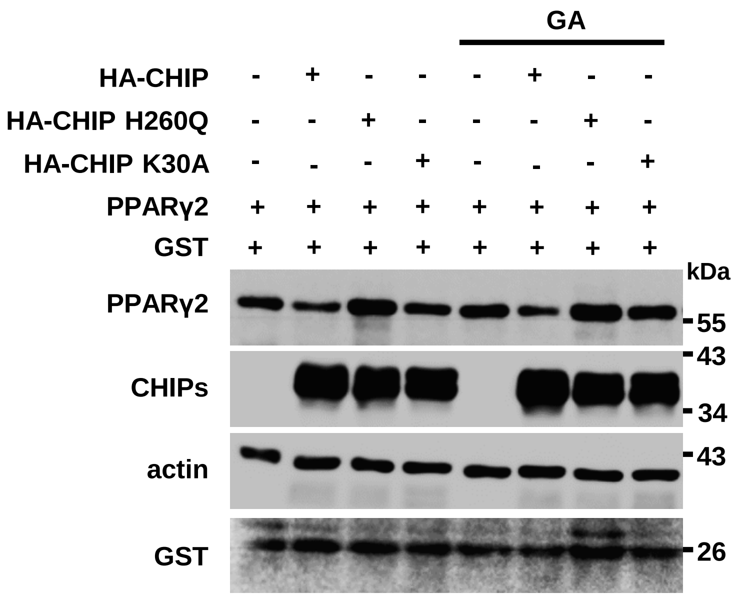
GA
HA-CHIP - + - - - + - -
HA-CHIP H260Q - - + - - - + -
HA-CHIP K30A - - - + - - - +
PPARγ2 + + + + + + + +
GST + + + + + + + +
kDa
PPARγ2
55
43
CHIPs
34
actin
43
GST
26
